# Three-dimensional region-based study on the relationship between soft and hard tissue changes after orthognathic surgery in patients with prognathism

**DOI:** 10.1371/journal.pone.0200589

**Published:** 2018-08-01

**Authors:** Lun-Jou Lo, Jing-Ling Weng, Cheng-Ting Ho, Hsiu-Hsia Lin

**Affiliations:** 1 Department of Plastic and Reconstructive Surgery, and Craniofacial Research Center, Chang Gung Memorial Hospital, Chang Gung University, Taoyuan, Taiwan; 2 Craniofacial Research Center, Chang Gung Memorial Hospital, Chang Gung University, Taoyuan, Taiwan; 3 Department of Craniofacial Orthodontics, Department of Dentistry, Chang Gung Memorial Hospital, Linkou, Taiwan; University Hospital Muenster, GERMANY

## Abstract

Both deep understanding and reliable prediction of postoperative soft tissue changes are crucial for planning orthognathic surgery. Instead of estimating soft tissue responses by measuring individual landmark changes, this study aimed to investigate the relationship (ratio) between soft and hard tissue movements in different facial regions through three-dimensional cone-beam computed tomography (CBCT). Preoperative and postoperative CBCT images were superimposed using the surface registration method on the basis of the cranial base, and 10 facial regions of interest were defined. Region-based volumetric subtractions between the preoperative and postoperative segments were performed. The volumetric differences and surface of each region were used to estimate the average movement. Correlation and regression analyses were performed to examine the relationships between the corresponding soft and hard tissue movements. An overall pattern of facial soft tissue movement was observed in patients with prognathism who underwent orthognathic surgery. The experiment results have shown that mean ratios for the average soft-to-hard tissue movements in the facial regions varied, which may not exactly be similar to the published reports because of the population biocharacteristics and study methods, but the trend is in agreement with the previous studies. Additionally, the prediction capability of the regression model was significantly high, ranging from 0.786 to 0.857, in upper lip, upper vermilion, and chin regions, thus demonstrating that the skin outline changes in these critical regions could be reliably predicted from the underlying bone movements. These results could likely be applied in future soft tissue simulation in orthognathic surgery.

## Introduction

Orthognathic surgery corrects maxillomandibular deformity associated with dental malocclusion through osteotomy and repositioning of the maxilla and mandible to achieve an ideal occlusal relationship and facial appearance. A change in the facial appearance relies on the underlying skeletal movement. Comprehensive understanding of the relationship between the bone movement and soft tissue response is crucial for predicting postoperative facial change and useful for treatment planning and patient consultation.

Published studies have investigated the relationship between the soft tissue profile change and the underlying skeletal movement by using two-dimensional (2D) cephalometric analysis [1–7]. A widely used method is to manually measure several selected landmarks on the hard and soft tissues in superimposed preoperative and postoperative images. However, numerous 2D approaches have extensively focused on the linear and angular measurements of certain facial landmarks in the mid-sagittal plane and have neglected the three-dimensional (3D) anisotropic behavior of soft tissues as well as the inherent limitations of the presentation of a complex 3D craniomaxillofacial structure in 2D [8–15].

3D images have been increasingly used in clinical settings to present complex facial structures composed of hard and soft tissues. In particular, cone-beam computed tomography (CBCT) captures both soft and hard tissues, and is becoming commonly used to assess surgical outcomes and to evaluate the 3D facial changes caused by orthognathic surgery according to the underlying bone movement [16–26]. In similar studies, 3D landmarks associated with the nose, upper and lower lips, and mandible have been selected to determine the correlations and proportions of soft-to-hard tissue changes in patients with class III malocclusion undergoing two-jaw orthognathic surgery [27–31]. However, previous methods have converted 2D cephalometric landmarks to a 3D cephalometric system but have not comprehensively addressed the 3D change. Recent advances in virtual surgical planning combined with commercial software opened up new possibilities in predicting facial soft tissue changes following orthognathic surgery based on the mass sprung model, finite element model, and mass tensor mode algorithms. However, the prediction accuracy in critical regions such as the lips, and chin is required to improve [32,33].

Prognathism with class III dental malocclusion is a common facial deformity, requiring orthognathic surgery for correction. The estimation and prediction of the facial outcome is imperative for both clinicians and patients. However, the use of commercial software has its limitations, especially in predicting soft tissue profile changes, since the default relationship (ratio) of soft to hard tissue movements is not realistic. This study aimed to investigate a general trend of relationship (ratio) of the facial soft tissue changes with the underlying skeletal movement in the designated facial regions of interest after two-jaw orthognathic surgery for patients with prognathism through 3D CBCT.

## Materials and methods

### Ethics statement

This retrospective study was conducted and approved by Chang Gung Craniofacial Center, Taiwan. All experiments were performed with the approval of the Institutional Review Board (IRB) of Chang Gung Memorial Hospital (IRB Nos.: 100-2842B and 103-6475C) and the study methods were carried out in accordance with the approved guidelines of the IRB. Written informed consent documents were obtained from the patients or from the guardians of patients younger than 20 years. The patient depicted in the Figures provided informed consent for the publication of his medical images (S1 File).

### Patient collection

This study was based on CBCT images of 24 selected patients (8 females and 16 males) enrolled from the Craniofacial Center, Chang Gung Memorial Hospital. The patient age was 18–35 years, with a mean age of 24.0 ± 4.8 years at the time of surgery. The inclusion criteria comprised the skeletal maturity of the patients prognathism with class III malocclusion, and the status of undergoing two-jaw orthognathic surgery. In this study, facial asymmetry was defined as absolute distance difference between both U6 (the most inferior point of the mesiobuccal cusp of each first upper molar in the profile plane.) perpendicular to the FH plane on each side being equal to or larger than 2 mm. Four patients (case no. 5, 10, 19, 21) were found to have facial asymmetry [34]. The angle ANB is the difference between SNA and SNB representing the relative position of the maxilla to the mandible. The mean and standard deviation of preoperation and postoperation ANB were -4.24 ± 2.15 and 2.57 ± 1.86, respectively. Patients with a cleft lip, cleft palate, or other craniofacial anomalies, major medical diseases, regular administration of medication, a history of facial trauma, degenerative or inflammatory conditions, previous orthognathic surgery, or inadequate imaging were excluded. All patients were treated with maxillary advancement and posterior impaction through LeFort I osteotomy and mandibular setback through BSSO to achieve a normal dentoskeletal relationship. The extent of skeletal movement achieved using the treatment plan was 1–3 mm for the maxillary advancement, 2–5 mm for the maxillary posterior intrusion, and 3–12 mm for the mandibular setback. Genioplasty was performed in 13 patients, with a movement of 1–8 mm for the advancement.

### Image acquisition

3D maxillofacial images of patients were acquired using an i-CAT CBCT scanner (Imaging Sciences International, Hatfield, PA, USA). The extended field of view was 22 cm (height) × 16 cm (depth), 120 kV, 5 mA, 50 Hz for a scanning time of 40 seconds and a voxel size of 0.4 × 0.4 × 0.4 mm. The CBCT scans were obtained within 1 month preoperatively and at a minimum of 9 months postoperatively when the brackets had been removed. During image acquisition, the patients were asked to relax their facial musculature, keep their eyes closed, and place the teeth in habitual occlusion. Patient data were stored in the digital imaging and communications in medicine (DICOM) format and subsequently imported to SimPlant Pro (Materialize Dental, Leuven, Belgium). Threshold segmentation was used for 3D model reconstruction of data obtained from DICOM files by identifying and delineating the anatomical structures of interest in the CBCT images. The resulting CBCT models were 3D representations of soft or hard tissues (bones and teeth) of each patient by defining a range of threshold values (Hounsfield units, HU). Threshold values of 300–400 HU and 800–900 HU were selected to identify the hard and soft tissues, respectively. The 3D models were subsequently exported to stereolithography (STL) files for registering preoperative and postoperative images.

### Image registration

**Fig 1:**
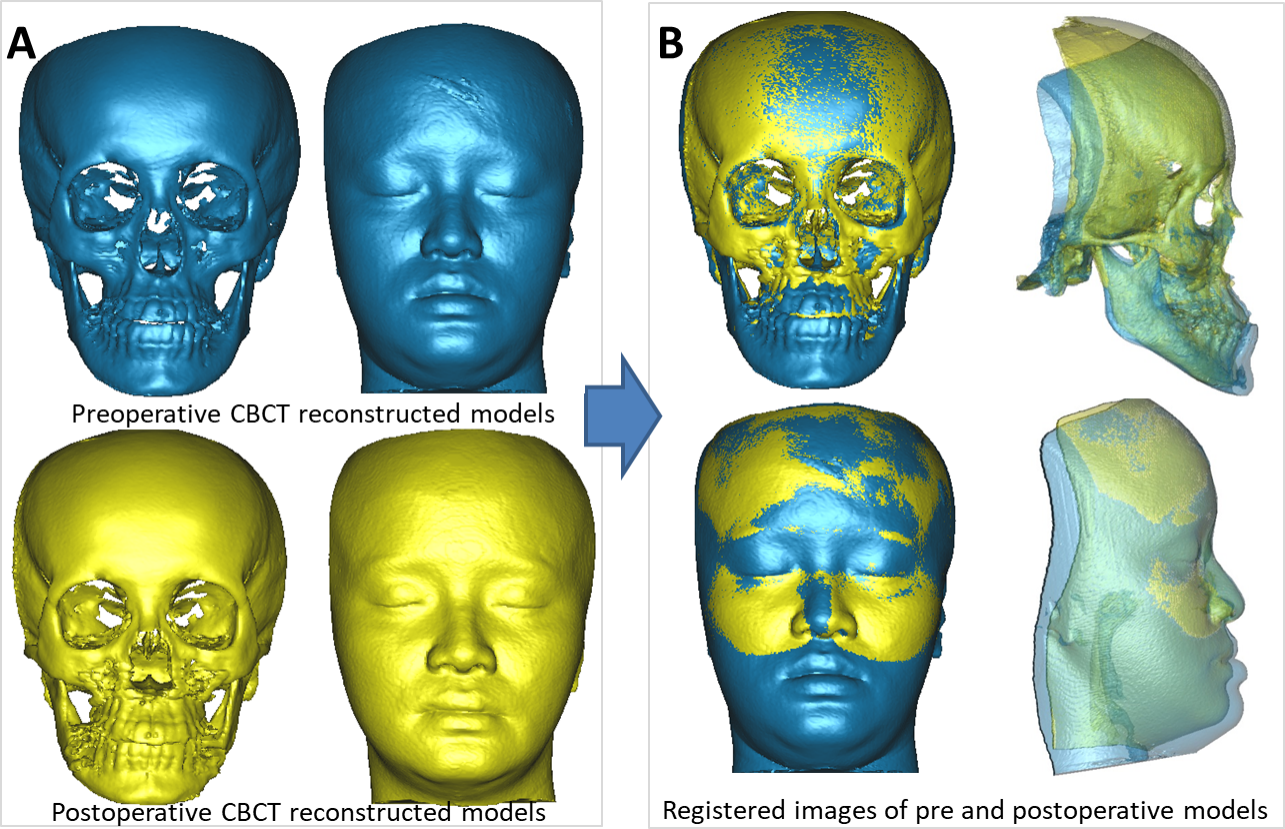
Cranial base registration of preoperative and postoperative CBCT images (case no. 21). 3D preoperative and postoperative models (**A**). Registration of preoperative and postoperative CBCT models (**B**).

**Fig 2:**
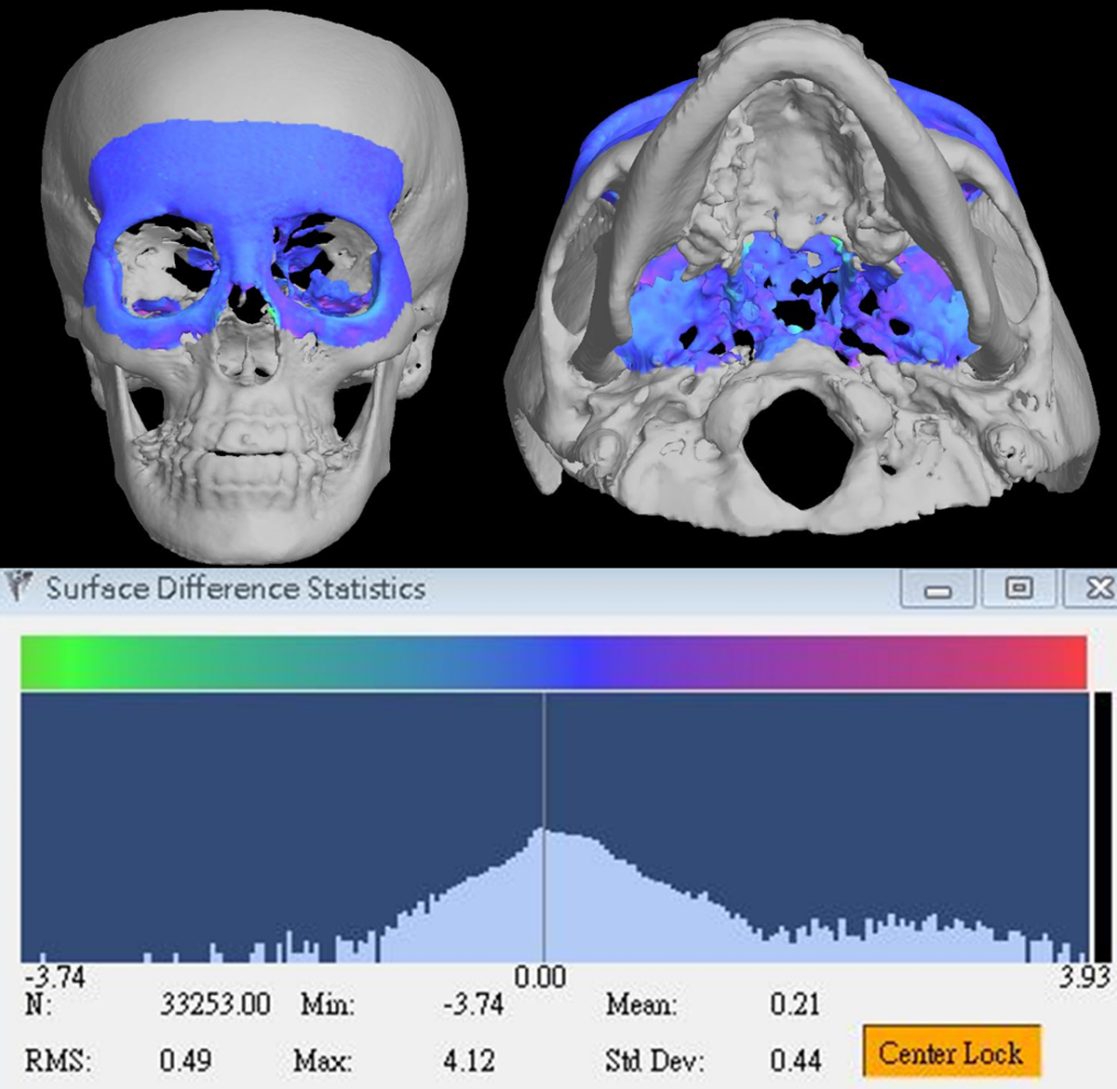
Accuracy of the cranial base registration in the color map of distance between the registered pre- and postoperative CBCT images (case no. 21).

To evaluate the soft tissue changes associated with hard tissue movements, the first step was to determine the volumetric changes in facial images caused by the orthognathic surgery. The preoperative and postoperative CBCT models were superimposed using the surface registration method on the basis of the cranial base, which was stable and unaffected by the surgery (Fig 1, case no. 21) [35]. The accuracy of the cranial base registration was verified by observing the distance color map in terms of the root-mean-square deviation (RMSD) between the registered preoperative and postoperative images (Fig 2, case no. 21). The RMSD errors between the registered surfaces ranged from 0.38 to 0.49 mm (with a mean of 0.41 mm) for the 24 patients. The deviation value was automatically calculated, and any value of 0.5 mm or less was considered acceptable to ensure that the corresponding reference areas had the maximum possible accuracy [36,37]. After the registration, 3D preoperative and postoperative models for soft and hard tissues were subtracted, resulting in volumetric differences for further measurement.

### Defining the facial regions of interest

**Fig 3:**
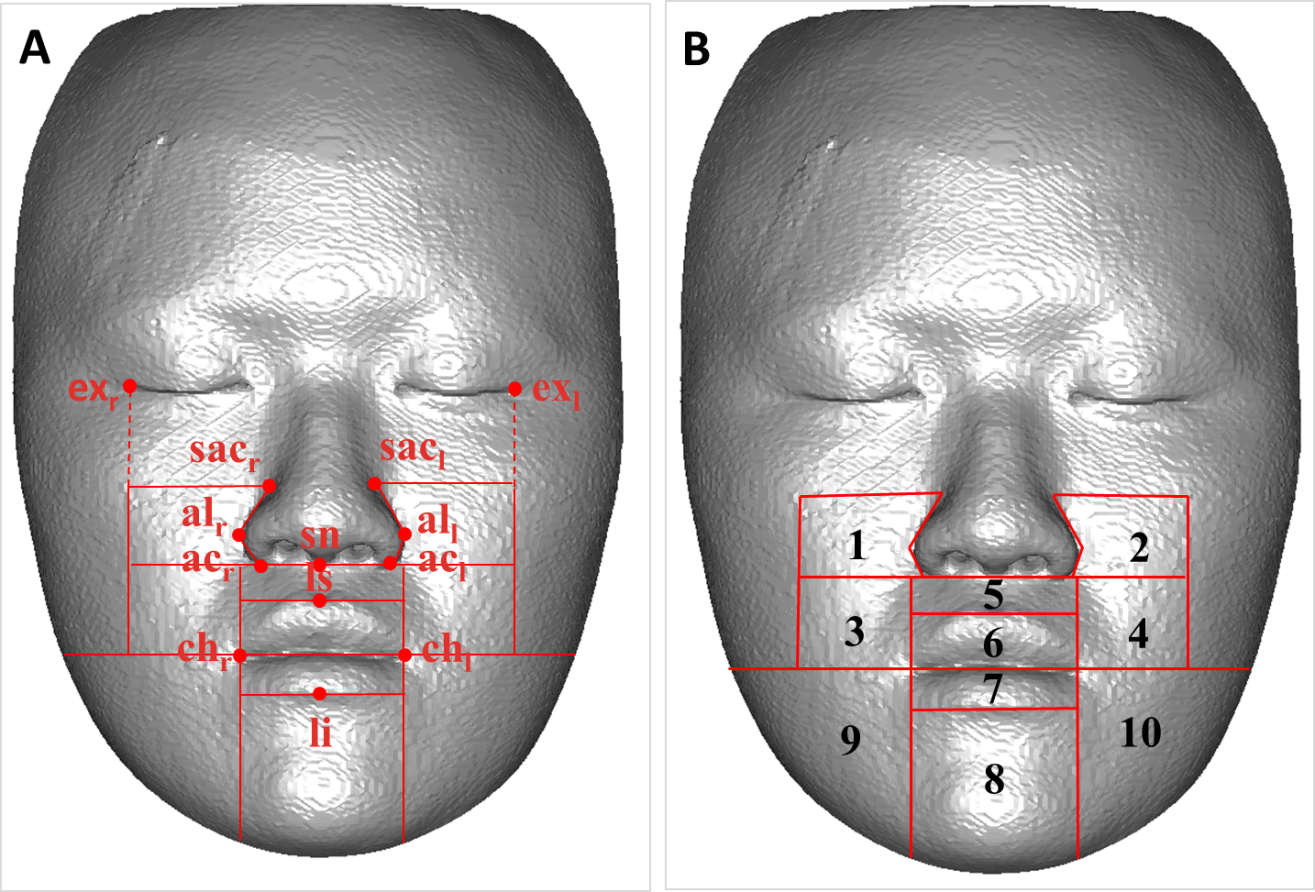
Definition of face regions of interest. The identification of cephalometric landmarks in the 3D soft model and the border of face regions (**A**). The frontal view of 10 isolated face regions (**B**).

Published studies have reported that soft tissue changes depend on several variables related to the facial structures, and the change are different in various facial regions; therefore, dividing the maxillofacial space into several subunits for practical examination is imperative [38]. First, a hard tissue-based reference system was developed and used for adjusting the head position of the registered images [39]. Subsequently, 10 separated regions of interest were defined and constructed on the basis of specific anatomical landmarks on the preoperative soft tissue surface to investigate the changes in each region. The regions were divided according to the original facial esthetic unit theory stated by Gonzles-Ulloa [40]; however, modifications were conducted in this study. Exocanthion (ex_l_ and ex_r_) and cheilion (ch_l_ and ch_r_) landmarks were indicated; they formed the borders parallel to the vertical plane of the reference frame. Lateral landmarks were indicated as the superior alar curvature (sac_l_ and sac_r_), alar curvature (ac_l_ and ac_r_), and cheilion and central landmarks were indicated as the subnasale (sn), labiale superior (ls), and labiale inferius (li); they formed borders parallel to the horizontal plane of the reference frame (Fig 3). Lateral areas outside the border of exocanthion and above the cheilion line were not computed because the underlying hard tissue was small and inappropriate for movement measurement. Table 1 lists the definitions of the cephalometric landmarks in the 3D soft tissue model, and Table 2 presents the definitions of the 10 isolated facial regions of interest. The nasal zone had different change patterns after orthognathic surgery and therefore was excluded from this study [41].

**Table 1:** Definition of 3D soft tissue landmarks.

Landmark	Abbreviation	Definition
**Exocanthion**	ex_l_, ex_r_	The leftmost and rightmost points at the outer commissure of the eye fissure
**Superior alar curvature**	sac_l_, sac_r_	The leftmost and rightmost points at the upper margin of the curved base line of each alar
**Alare**	al_l_, al_r_	The most lateral point on each alar contour
**Alar curvature**	ac_l_, ac_r_	The most lateral point in the curved base line of each ala, indicating the facial insertion of the nasal wing base
**Subnasale**	sn	The midpoint on the nasolabial soft tissue contour between the columella crest and the upper lip
**Labiale superius**	ls	The midpoint of the upper vermilion line
**Labiale inferius**	li	The midpoint of the lower vermilion line
**Cheilion**	ch_l_, ch_r_	The leftmost and rightmost points located at each labial commissure

**Table 2:** Definition of the 10 isolated face regions of interest.

Region no.	Region	Definition
**1**	Right paranasal region	Region of the right cheek adjacent to the ala of the nose
**2**	Left paranasal region	Region of the left cheek adjacent to the ala of the nose
**3**	Right supracommissural region	Right anterior cheek region adjacent to upper lip, inferior to ala and above oral commissure
**4**	Left supracommissural region	Left anterior cheek region adjacent to upper lip, inferior to ala and above oral commissure
**5**	Upper lip region	Region below the nose and above the upper vermilion
**6**	Upper vermilion region	Vermilion region of the upper lip
**7**	Lower vermilion region	Vermilion region of the lower lip
**8**	Chin region	Region of chin between both commissures
**9**	Right infracommissural region	Right lower facial region below the oral commissure and lateral to the chin
**10**	Left infracommissural region	Left lower facial region below the oral commissure and lateral to the chin

### Computing skin and bone movements in the defined facial regions

**Fig 4:**
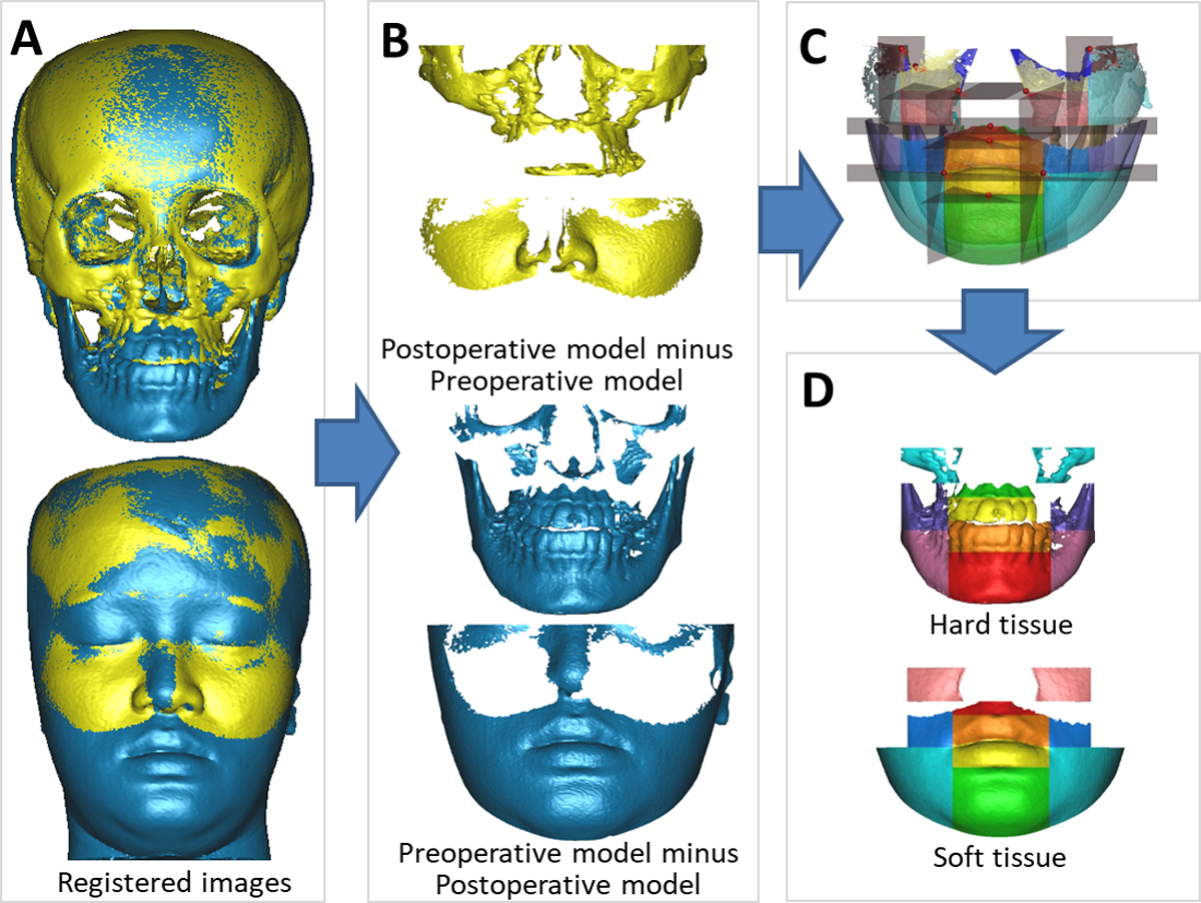
Volumetric difference of isolated soft and hard tissue regions. Registered CBCT models (**A**), the volumetric differences after orthognathic surgery (**B**), the overall volumetric difference divided into 10 isolated regions based on the constructed planes (**C**), and 10 isolated regions of soft tissue and hard tissue (**D**).

The volumetric difference was measured on the lower half of the face, which was altered by the orthognathic surgery. After superimposition, the volume of the 3D preoperative image was subtracted from that of the 3D postoperative image, resulting in an overall volumetric difference of the backward movement because of mandibular setback. To obtain the overall volumetric difference of the forward movement because of the maxillary advancement, the volume of the postoperative 3D image was subtracted from that of preoperative 3D image. The overall volumetric difference was divided into the 10 defined regions. The volumetric changes in soft and hard tissues in each region were subsequently exported as STL files for further evaluation (Fig 4).

**Fig 5:**
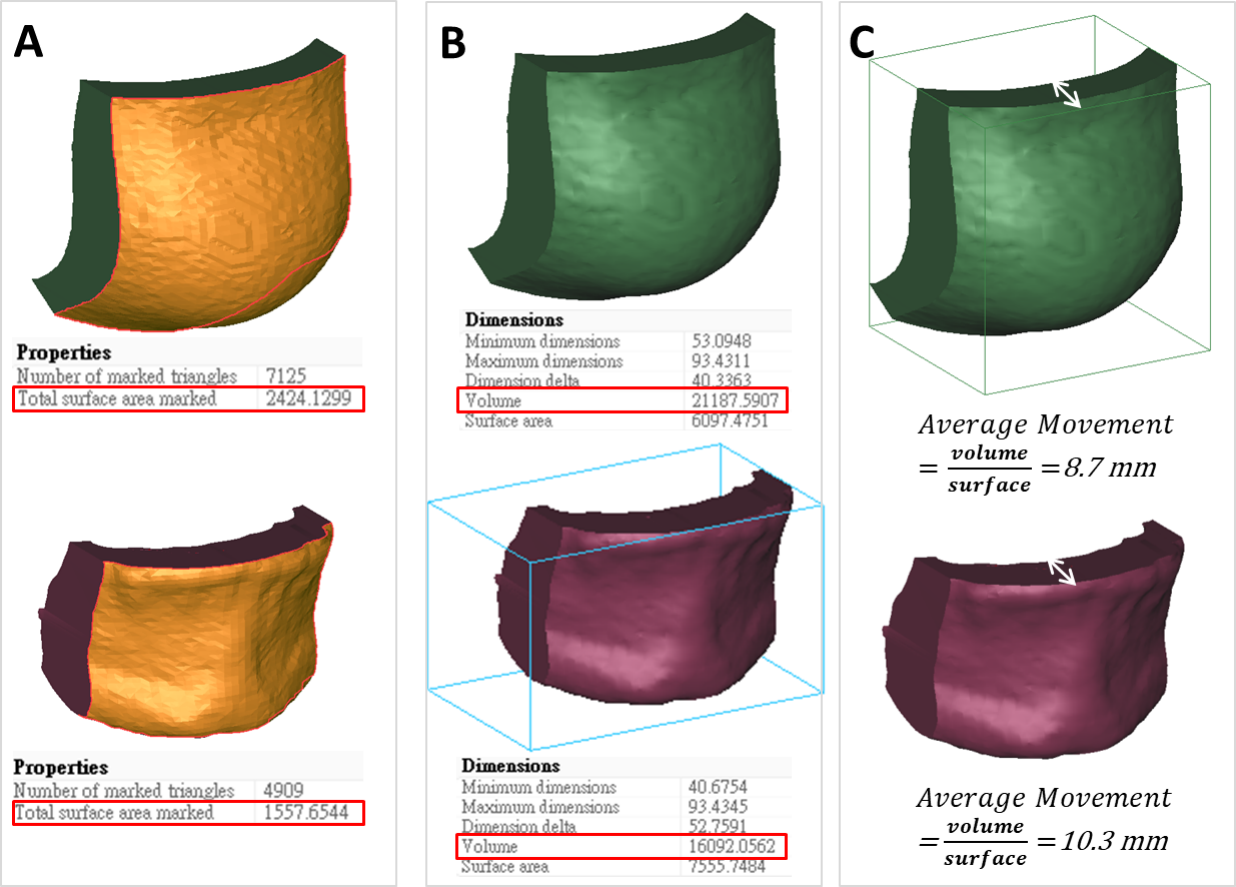
Volumetric difference and average movement of soft and hard tissue for chin region. The model of volumetric change (**A**), volumetric difference (**B**), and average movement (**C**).

The value of volumetric change (mm^3^) and surface area (mm^2^) of preoperative images for each facial region were automatically computed using 3-matic software (Materialize Dental). The average movement (mm) of soft (skin) or hard tissues (the underlying bone) in each facial region was defined as the volume divided by the preoperative surface area. The area defined in the preoperative image surface was used for calculating the movement of the postoperative surface area of each region (Fig 5). The hypothesis of this study was that all point in the same region is in a uniform movement distance.

### Statistical analysis

The ratios of the average soft and hard tissue movements were evaluated for the 10 facial regions of all patients. Scatter plots were used to investigate the relationship of the soft tissue changes with the underlying hard tissue movements, and the Pearson correlation coefficient was calculated for each corresponding pair. Regression analysis was used to evaluate the associations between the average soft and hard tissue movements, resulting in a coefficient of determination (denoted by R squared, R^2^). Statistical significance of the overall fit was determined using the F-test, followed by t tests for individual parameters. Statistical significance was established at 95% confidence intervals (*P* < .05). Statistical analyses were performed using SPSS (released in 2008, SPSS statistics for Windows, Version 17.0, SPSS Inc, Chicago, USA).

## Results

**Fig 6:**
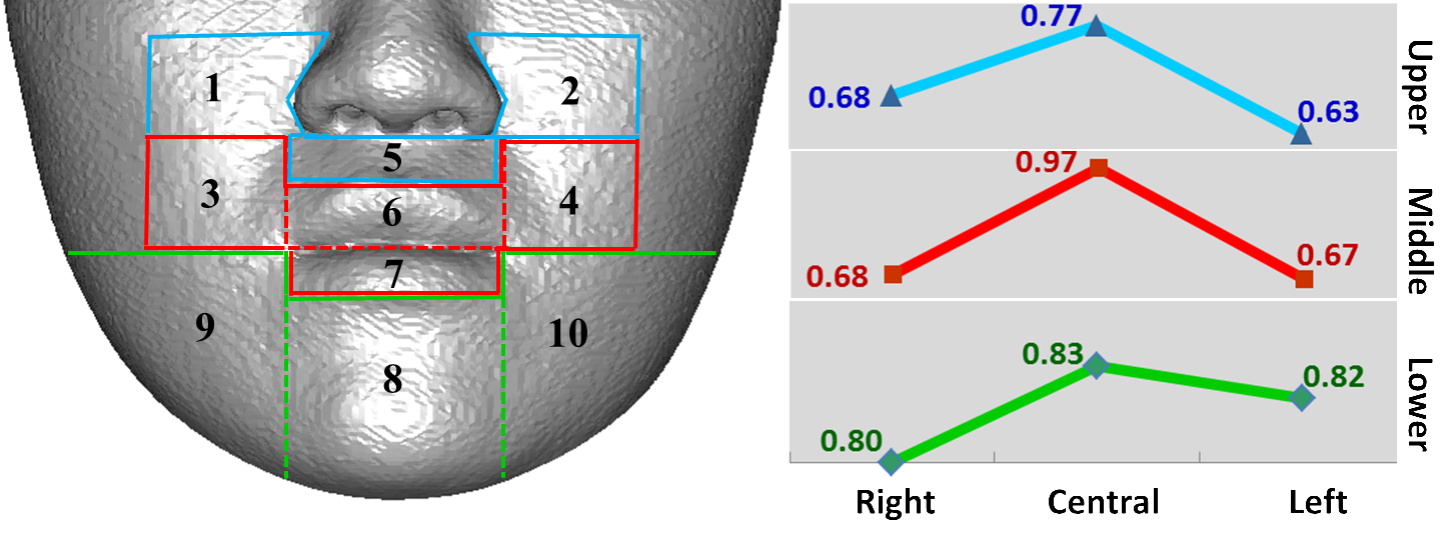
Horizontal trend of average movement ratio of 10 regions.

**Fig 7:**
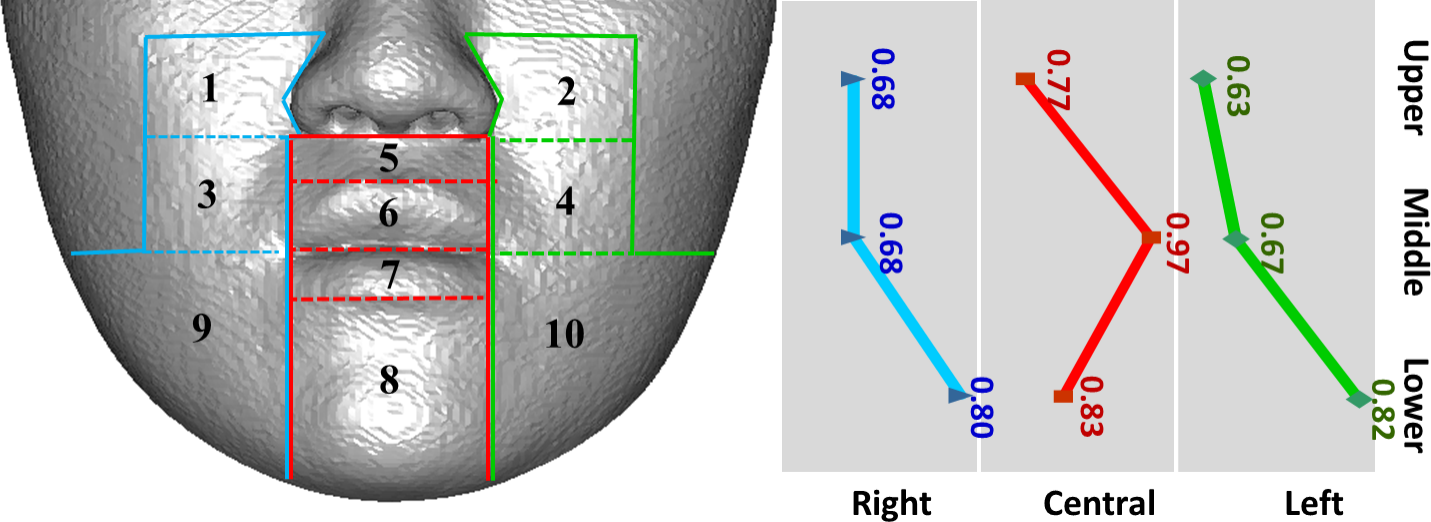
Vertical trend of average movement ratio of 10 regions.

Tissue changes in males and females were not considerably different; therefore, the data were combined for analysis. Table 3 presents the mean and standard deviation of the ratio of the average soft to hard tissue movements in each region. For further demonstration, the 10 regions were classified into three horizontal sections on the basis of the location for LeFort I osteotomy or BSSO: upper, middle, and lower (Fig 6). The regions were also classified into three vertical sections according to the distance from the midsagittal plane: right, central, and left (Fig 7). The results revealed a tendency of the mean ratio in the central region to be larger than that in the lateral regions for all horizontal sections (Fig 6). The mean ratio increased from upper to lower regions, except in the central section (Fig 7).

**Table 3:** Average volumetric difference, the average surface, and average calculated movement of 24 patients in relevant face region. Although the paranasal and upper lip region are forward, other areas are mostly backward.

Region no.	Region	Volumetric difference (mm^3^)		Preoperative region surface (mm^2^)		Average movement (mm)		Average movement ratio
		Hard tissue	Soft tissue	Hard tissue	Soft tissue	Hard tissue(H)	Soft tissue(S)	S/H ratio(%)
**1**	Right paranasal	485±146	476±324	355±84	506±209	1.4±0.4	0.9±0.4	0.68±0.36
**2**	Left paranasal	464±160	399±280	363±102	463±189	1.3±0.3	0.8±0.3	0.63±0.33
**3**	Right supracommissural	-1899±850	-954±688	833±349	535±181	-2.5±1.5	-1.6±0.8	0.68±0.20
**4**	Left supracommissural	-1918±1031	-1039±720	796±398	594±155	-2.6±1.5	-1.6±0.9	0.67±0.34
**5**	Upper lip	119±613	-65±520	460±130	350±180	0.3±1.2	0.0 ±1.2	0.77±0.35
**6**	Upper vermilion	-681±835	-799±748	539±189	563±176	-1.2±1.3	-1.3±1.2	0.99±0.48
**7**	Lower vermilion	-3533±1293	-3732±1314	622±180	697±169	-5.7±1.6	-5.4±1.6	0.95±0.19
**8**	Chin	-12597±4263	-14503±5572	1586±291	2211±506	-8.0±2.4	-6.5±2.1	0.83±0.11
**9**	Right infracommissural	-6580±3706	-10994±4145	1677±417	3357±679	-4.2±1.2	-3.2±0.9	0.80±0.23
**10**	Left infracommissural	-7464±3463	-12416±5485	1682±451	3406±742	-4.3±1.2	-3.6±1.0	0.82±0.19

Positive values of the surface distance indicate displacement in the forward direction and negative values indicate displacement in the backward direction.

S/H ratio is the ratio of soft tissue movement to hard tissue movement

**Fig 8:**
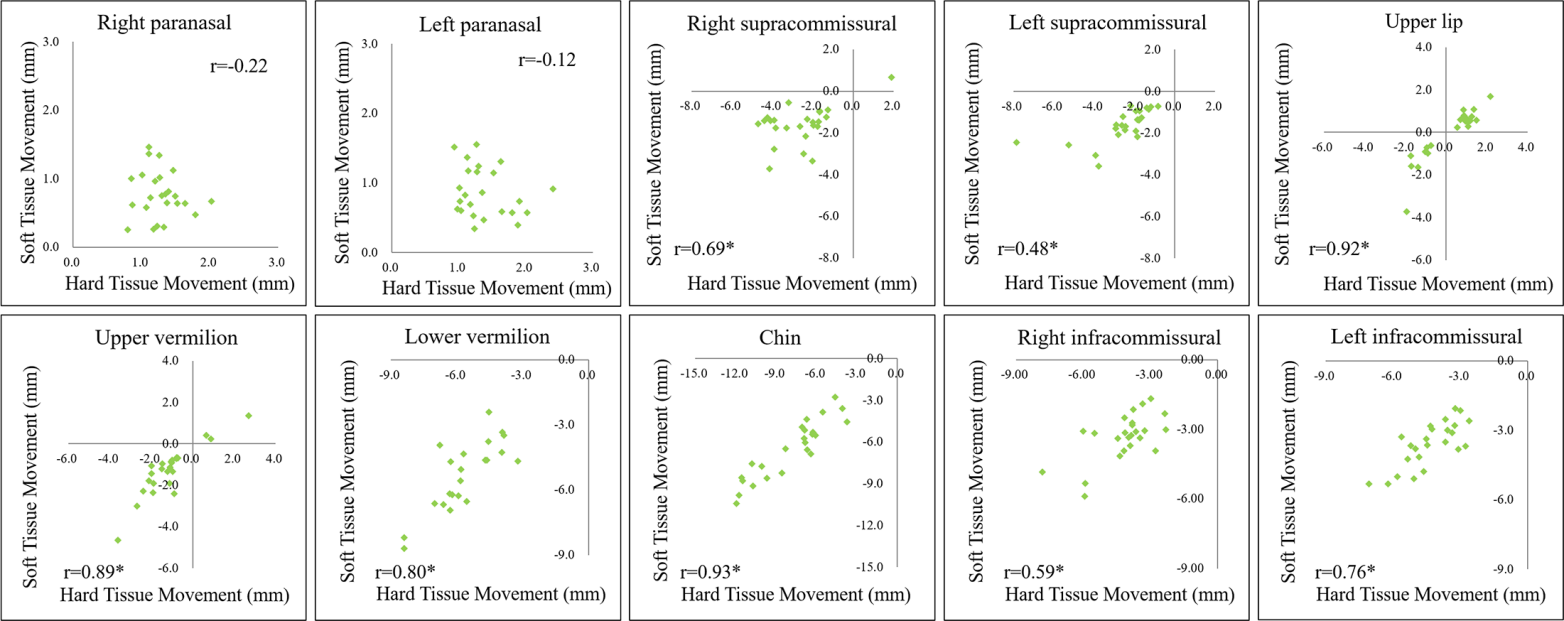
Scatter plots of the soft tissue average movements with respect to underlying hard tissue average movements for 10 regions; r denotes the correlation coefficient for each plot.

The scatter plots of the 10 regions showed linear relationships between the soft and hard tissue movements; each the correlation coefficient is denoted by r (Fig 8). The results revealed extremely strong correlations among upper lip, upper vermilion, lower vermilion, and chin regions (r ≥ 0.8); strong correlations between right supracommissural and left infracommissural regions (0.6 < r < 0.8); moderate correlations between left supracommissural and right infracommissural regions (0.3 ≤ r ≤ 0.6); and weak or no correlation between right and left paranasal regions.

Table 4 displays the results of linear regression analysis. The results showed that high R^2^ values in the upper lip, upper vermilion, and chin regions, ranging from 0.786 to 0.857, were associated with statistical significance. The results revealed that the regression models could be used to predict the soft tissue movement from the corresponding hard tissue movements. For other regions, R^2^ values were lower, ranging from 0.229 to 0.641, indicating that the regression models had weak prediction capabilities in these regions. These regression models could not be used to accurately predict the soft tissue movements from the corresponding hard tissue movements.

**Table 4:** Linear regression model of the soft tissue average movements and underlying hard tissue average movements for the regions.

Region no.	Region	Regression model	Coefficient of determination (R^2^)	Significance level (*P*)
**1**	Right paranasal	y = -0.206x+1.152	0.048	0.306
**2**	Left paranasal	y = -0.150x+0.963	0.015	0.568
**3**	Right supracommissural	y = 0.359x – 0.697	0.476	0.000
**4**	Left supracommissural	y = 0.304x – 0.837	0.229	0.018
**5**	Upper lip	y = 0.901x – 0.279	0.848*	0.000
**6**	Upper vermilion	y = 0.843x – 0.331	0.786*	0.000
**7**	Lower vermilion	y = 0.824x	0.641	0.000
**8**	Chin	y = 0.788x	0.857*	0.000
**9**	Right infracommissural	y = 0.441x –1.440	0.353	0.002
**10**	Left infracommissural	y = 0.646x	0.581	0.000

x denotes the soft tissue average movement.

y denotes the hard tissue average movement.

R^2^<0.5 is meaningful, R^2^ = 0.7 may be interpreted as follows: 70% of the variance in the response variable can be explained by the explanatory variables. The remaining thirty percent can be attributed to unknown factor.

*: *P* ≤ 0.05 statistically significant, and if *P* > 0.05, that hypothesis must be rejected and the alternative hypothesis must be accepted as true.

To calculate the effective sample size, a power analysis for linear regression was conducted using G*Power 3.1.6 (Heinrich-Heine-Universität Düsseldorf, Düsseldorf, Germany). The analysis indicated that at least 23 samples would be required for an α of 0.05 and a power of 0.7, showing that our study sample size was adequate.

## Discussion

Patients are sensitive to any changes in facial esthetics after orthognathic surgery. Therefore, highly reliable prediction of the soft-tissue results of surgery plays a principal role in improving treatment planning and patient–doctor communication. Soft tissue changes occur because of the underlying skeletal movements. The evaluation and prediction of soft tissue responses following orthognathic surgery have been widely studied. However, 2D cephalometric analysis has inherent limitations regarding the presentation of complex 3D changes in hard and soft tissues [8–14]. Recently, soft tissue responses to underlying skeletal changes have been evaluated using 3D imaging technology, such as 3D landmarks, color mapping, and volumetric difference [15, 17–20]. However, these results did not completely represent the 3D relationship between the corresponding soft-to-hard tissue changes in various face subunits [42–46]. 3D CBCT imaging is suitable for evaluating the facial soft tissue changes caused by orthognathic surgery according to the underlying bone movements because CBCT simultaneously displays both soft and bone tissues. In this study, a source of bias is whether orthodontic appliances affect the appearance of the soft tissues, particularly the position of the lips. Based on some previous quantitative studies, the changes in the perioral soft tissues after appliance removal are not clinically significant, but individual variations do exist. Thus, the effect of orthodontic brackets removal on lip positions was not considered relevant in this study [47–48].

### Mean ratio of the average soft to hard tissue movement

An overall trend was observed. Soft tissue changes followed hard tissue movements. The results revealed that the average movements of soft tissues were smaller than those of hard tissues, and ratios varied for different regions (Table 3). Our mean ratios may not exactly be similar to previously reported values because of the population biocharacteristics and study methods, but the trend is in agreement.

The mean ratios of the average movements of soft-to-hard tissues revealed that the values in central regions were higher than those in lateral regions for all horizontal sections (Fig 6), which was in accordance with previous 3D studies [26–31, 38, 42–44]. This observation explains why the soft tissues in the central regions are thinner and the adherence to the underlying bone is firm and less flexible. Therefore, the soft tissues in the central regions follow the underlying hard tissue movements more closely than do the soft tissues in the lateral regions, which are relatively loose and thick. Through laser imaging, Soncul et al. evaluated soft tissue changes and reported that the amount of soft tissue changes relative to hard tissue movements gradually decreased toward the lateral aspect after surgical correction of class III deformity [38]. Furthermore, Kim et al. used 121 representative points and revealed a similar pattern in patients with mandibular setback, stating that the farther the setback was located, the smaller was the movement ratio of the soft tissue [26].

Considering all vertical sections, the mean ratios of the average soft-to-hard tissue movements increased from upper to lower regions (Fig 7). This finding was similar to the finding of 2D cephalometric studies conducted using the midline landmarks; however, our ratios were unlike those of the 2D studies because our study used 3D region-based measurements [7, 9, 49].

### Relationship between ratios of the average soft to hard tissue movements

The scatter plots showed extremely strong relationships among the upper lip (r = 0.92), upper vermilion (r = 0.89), lower vermilion (r = 0.80), and chin (r = 0.93) regions. The results of regression analysis supported significant linear relationships of the average soft-to-hard tissue movements corresponding to the upper lip (R^2^ = 0.848), upper vermilion (R^2^ = 0.786), and chin (R^2^ = 0.857) regions. This finding provides evidence for using linear regression equations for predicting the average soft-to-hard tissue movements in these regions. Except for the lower vermilion region, results of all regions were in concordance with previous 2D cephalometric landmark studies [11, 50, 51], which reported linear relationships between soft-to-hard tissue changes in the maxillary incisor tip–labrale superius, mandibular incisor tip–labrale inferius, ponit B–mentolabial sulcus, and hard pogonion–soft pogonion over a wide range of skeletal changes [11]. Koh et al. indicated that when using a computer-assisted simulation system for orthognathic surgery, prediction errors are likely to be caused by the underestimation of the vertical position and overestimation of the horizontal position of the lower lip for bimaxillary surgery in patients with class III malocclusion50. Barakat et al. reported larger prediction differences in the lower lip and postulated that the reasons for the discrepancy were lip tonicity, length, posture, and mass [51].

As shown in Table 4, the linear relationships were significant among the right supracommissural (R^2^ = 0.476, *P* = .000), left supracommissural (R^2^ = 0.229, *P* = .018), lower vermilion (R^2^ = 0.641, *P* = .000), right infracommissural (R^2^ = 0.353, *P* = .002), and left infracommissural (R^2^ = 0.581, *P* = .000) regions. In these regions, the prediction capability was not consistent for estimating the overlying soft tissue changes. In right and left paranasal regions, the soft tissue moved with the bone tissue; however, the linear relationship was not significant for prediction. Altogether, these results might have been caused by the sample size and population biocharacteristics. Additional studies are required to analyze more patients and develop novel imagingmethods.

One limitation derived from the retrospective study design, patients CBCT scans were acquired at a minimum of 9 months postoperatively in order to avoid the surgical swelling. The predicted result should not be the same as the actual postoperative outcome due to the variance between surgical changes and the individual changes caused by skeletal relapse and postoperative orthodontics. Instead of estimating soft tissue responses by measuring individual landmark changes, our study aimed to find a general trend of relationship (ratio) between soft and hard tissue movements for various facial regions and provide a detailed information that can help to guide surgical planning. Nevertheless, this tolerance could be ignored. Additionally, the ratios can be effectively applied to commercial software as the parameters for orthognathic surgery simulation in predicting 3D soft tissue profile changes (S1 Fig). Using commercial software tools, the prediction of postoperative soft tissue movement values were converted to a vector diagram as shown in S2 Fig, and the magnitude, direction, and location of the postoperative soft-tissue movement in a color-scale plot was automatically displayed. As can be seen in S1 Fig, we can find the movement of the lateral area (1,2,3,4,9,10) has stronger curvature than center area (5, 6, 7, 8).

Predicting soft-to-hard tissue changes is difficult, however, our study provides comprehensive evidence from the linear regression model for predicting such movements, and the prediction is consisted to the upper lip, upper vermilion, and chin regions. The results focused on the skin profile changes in above-mentioned critical regions, which could be reliably predicted from the underlying bone movements. However, the experience of providing a more realistic soft tissue prediction for all regions will be collected for our future study, and the method still requires improvement for in the further.

## Supporting information

S1 File This is patient's agreement for the publication of his medical images. (PDF)

S1 Fig The ratios resulted from our study are applied to commercial software as the parameters for orthognathic surgery simulation in predicting 3D soft tissue profile changes. (TIF)

S2 Fig The prediction of postoperative soft tissue changes is converted to a vector diagram, and the magnitude, direction, and location of the postoperative soft-tissue movement in a color-scale plot was automatically displayed. (TIF)
