# Supplementary material for: Three-dimensional region-based study on the relationship between soft and hard tissue changes after orthognathic surgery in patients with prognathism
Source: PLoS One. 2018 Aug 1;13(8):e0200589. doi: 10.1371/journal.pone.0200589 (PMC6070212; doi:10.1371/journal.pone.0200589)
Supplement: S1 File — (PDF) [file pone.0200589.s001.pdf]

患者姓名
Patient Name 陳聖雯

病歷號碼
Registration No. 3958284

醫學影像同意書
(包括照相及 X 光)
Consent for Medical Imaging

我(們)同意長庚醫院顱顏中心應此次療程之病情評估、診斷、治療、手術、及後續追蹤需要，得以拍攝或錄製有關我/患者的相片、影帶或 X 光，並同意其作為治療醫師的檔案或病歷之部分。在不危害我(們)隱私的原則下，醫師可斟酌刊登這些照片、影帶或 X 光於醫學文獻或用於教育研究。其未經同意不得用於營利之途。此授權係出於自願不要求任何報償。

I (the patient) or I/We (the parent(s)/guardian(s) of the patient) do hereby authorize the Department of Craniofacial Center, Chang Gang Memorial Hospital (Dr. ____________________) to photograph, film, and/or videotape me/the patient during the course of evaluation, diagnosis, treatment (including surgical procedures), and follow-up; I/We understand this media may be part of the physician's files or medical record. I/We further consent to the use of such photographs, films, and/or videotapes in the medical literature and otherwise for educational or research purposes at the physician's discretion without liability to my/our privacy. The media may not be used for profit without my/our express permission. I/We understand that I/We will not be paid or rewarded for providing this authorization.

患者簽名
Patient signature: 陳聖雯

法定監護人簽名
Parent/guardian signature: ____________________

日期
Witness Date: 2010 / 11 / 10
