# Supplementary figures and images for: Three-dimensional region-based study on the relationship between soft and hard tissue changes after orthognathic surgery in patients with prognathism
Source: PLoS One. 2018 Aug 1;13(8):e0200589. doi: 10.1371/journal.pone.0200589 (PMC6070212; doi:10.1371/journal.pone.0200589)

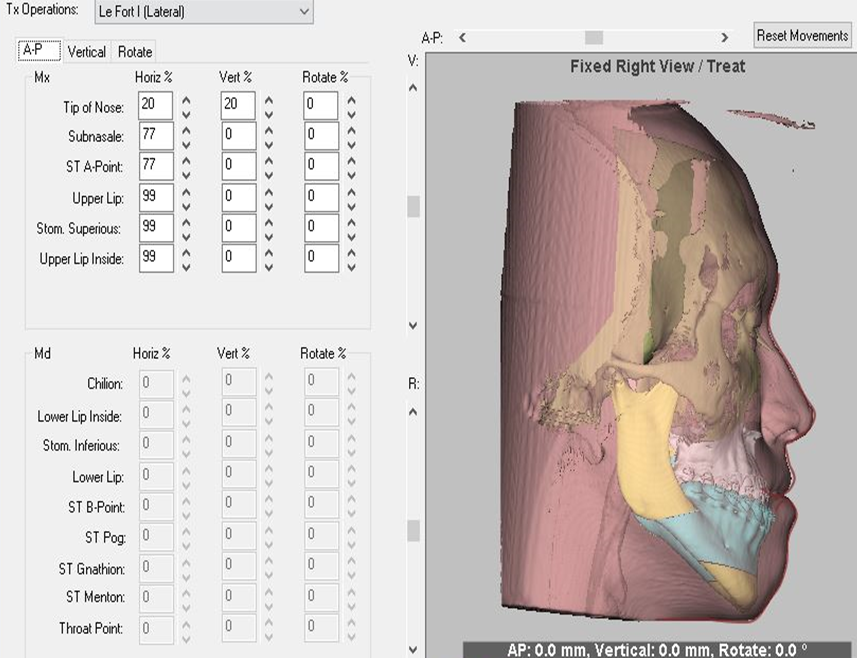

Supplement: S1 Fig — (TIF) [file pone.0200589.s002.tif]

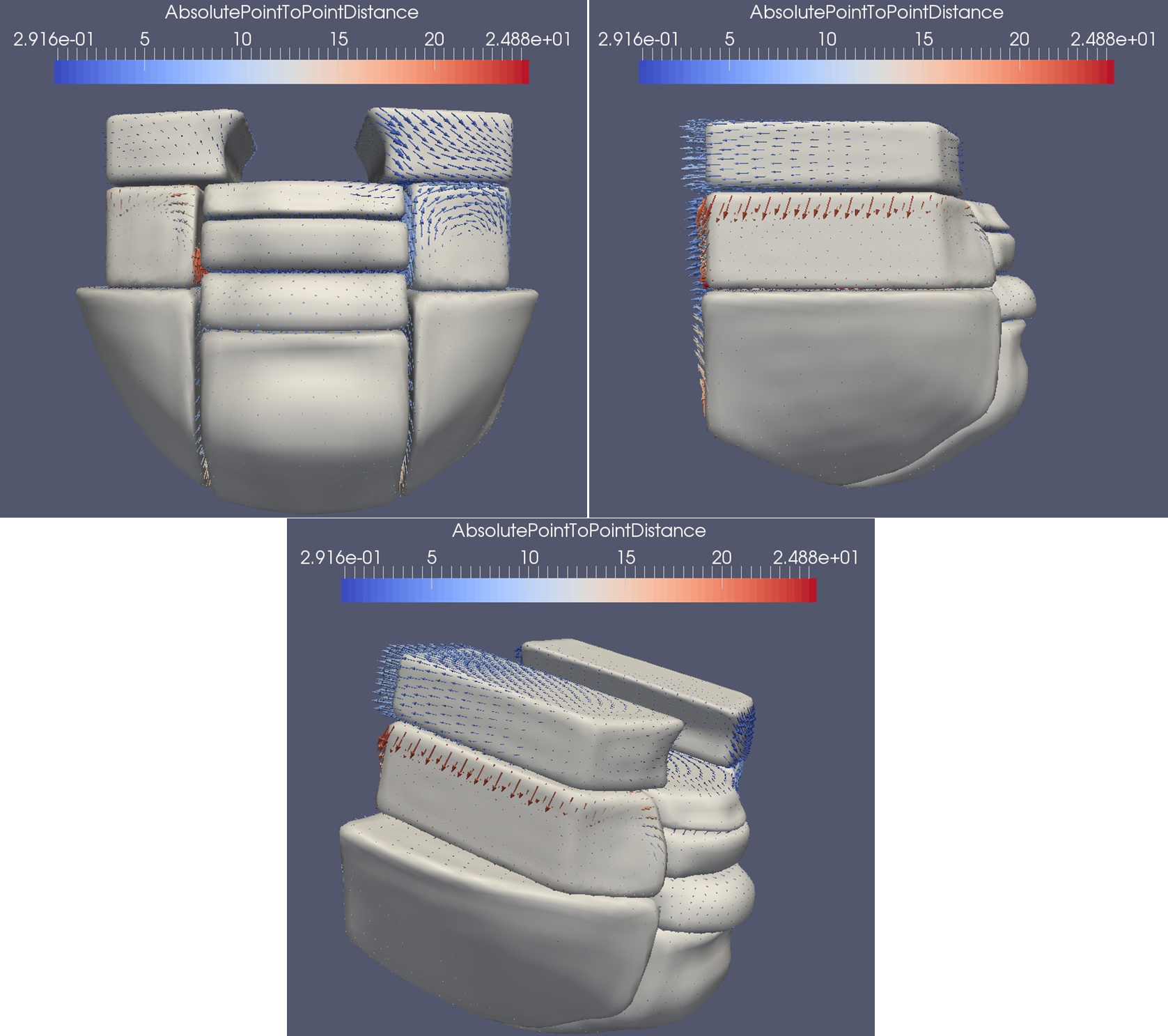

Supplement: S2 Fig — (TIF) [file pone.0200589.s003.tif]
